# A scoping review of wildfire smoke risk communications: issues, gaps, and recommendations

**DOI:** 10.1186/s12889-024-17681-0

**Published:** 2024-01-27

**Authors:** Morgan H. Vien, Susan L. Ivey, Hollynd Boyden, Stephanie Holm, Linda Neuhauser

**Affiliations:** 1 Health Research for Action, University of California Berkeley, School of Public Health, Berkeley, USA; 2 Western States Pediatric Environmental Health Specialty Unit, San Francisco, USA; 3 University of California, San Francisco, USA

**Keywords:** Risk communication, Wildfire smoke, Smoke exposure, Vulnerable populations, Communications dissemination, Health literacy

## Abstract

**Background:**

Wildfire smoke exposure has become a growing public health concern, as megafires and fires at the wildland urban interface increase in incidence and severity. Smoke contains many pollutants that negatively impact health and is linked to a number of health complications and chronic diseases. Communicating effectively with the public, especially at-risk populations, to reduce their exposure to this environmental pollutant has become a public health priority.

Although wildfire smoke risk communication research has also increased in the past decade, best practice guidance is limited, and most health communications do not adhere to health literacy principles: readability, accessibility, and actionability. This scoping review identifies peer-reviewed studies about wildfire smoke risk communications to identify gaps in research and evaluation of communications and programs that seek to educate the public.

**Methods:**

Four hundred fifty-one articles were identified from Web of Science and PubMed databases. After screening, 21 articles were included in the final sample for the abstraction process and qualitative thematic analysis. Ten articles were based in the US, with the other half in Australia, Canada, Italy, and other countries. Fifteen articles examined communication materials and messaging recommendations. Eight papers described communication delivery strategies. Eleven articles discussed behavior change. Six articles touched on risk communications for vulnerable populations; findings were limited and called for increasing awareness and prioritizing risk communications for at-risk populations.

**Results:**

This scoping review found limited studies describing behavior change to reduce wildfire smoke exposure, characteristics of effective communication materials and messaging, and communication delivery strategies. Literature on risk communications, dissemination, and behavior change for vulnerable populations was even more limited.

**Conclusions:**

Recommendations include providing risk communications that are easy-to-understand and adapted to specific needs of at-risk groups. Communications should provide a limited number of messages that include specific actions for avoiding smoke exposure. Effective communications should use mixed media formats and a wide variety of dissemination strategies. There is a pressing need for more intervention research and effectiveness evaluation of risk communications about wildfire smoke exposure, and more development and dissemination of risk communications for both the general public and vulnerable populations.

## Background

### Wildfire smoke events and their health impacts

Wildfire smoke exposure is a growing public health concern. Large wildfire events have increased [1] due to multiple factors including increased aridity and storms from climate change, and outdated fire suppression strategies. This increase has led to larger overall acreage burned and more smoke days per year [2]. Additionally, wildland urban interface (WUI) fires and homes in the WUI have increased [1, 3]. A growing body of research over the past two decades has documented that such smoke contains many pollutants that negatively impact health, including over the long-term [4]. Wildfire smoke is linked to adverse cardiovascular, respiratory, dermatologic [5–7] and nervous system outcomes [6–10]; has metabolic effects linked to diabetes [8]; and contains toxins that can contribute to cancer [11]. The evidence for mortality effects (respiratory and all-cause) is particularly robust [6, 10, 12–14]. Additionally, recent research demonstrates that smoke may contribute to adverse pregnancy and birth outcomes such as low birthweight [15, 16], infant wheezing [16], and infertility [17]. Other research also points to the psychiatric consequences of wildfire smoke [18, 19].

Wildfire smoke is especially problematic for children, contributing to the development of asthma and increasing asthma exacerbations [7, 16, 20–22]. Children are known to be especially vulnerable, both because they are growing and also because they breathe more pollutants relative to their size compared to adults [3, 23]. Recent reviews of wildfire smoke effects in children indicate a rapidly growing body of literature, with substantial evidence of respiratory, mental health, and birthweight effects in those exposed to wildfire smoke, in addition to some evidence for a variety of impacts on other conditions such as cardiac function [24–26]. Wildfire smoke has especially impacted vulnerable at-risk populations [2], including Black, Indigenous, and People of Color (BIPOC) [1, 27, 28] as well as rural farming communities [18]. Vulnerable adult populations are more likely to have several chronic conditions, such as diabetes and cardiovascular disease, which already impact certain populations more than others, e.g., Black/African Americans have more hypertension and stroke, certain Latino populations and Native Americans have higher risks for Type 2 diabetes [29]. Vulnerable populations are at higher risk for exacerbations of those conditions, such as experiencing myocardial infarctions and/or strokes, during wildfire smoke events [6, 9, 10]. Therefore, the need for effective risk communications regarding wildfire smoke is especially critical for these vulnerable populations.

### Current state of wildfire smoke risk communications

As the incidence and severity of wildfire smoke events increase and the serious health impacts become better understood, communicating effectively with the public to reduce their exposure to this environmental pollutant is a priority for public health interventions, especially for the most at-risk populations. Wildfire smoke risk communication research and communication interventions have greatly increased during the past decade [7, 30–32]. This includes messaging primarily by publicly-funded organizations such as the US Environmental Protection Agency (EPA), US Department of Health and Human Services, Centers for Disease Control and Prevention, and state, county, and city governments in the U.S [33–37]. as well as from community organizations in other countries [35, 38], all of which have prioritized risk communication on wildfire smoke related health effects.

There are a number of concerns with existing risk communication materials. Wildfire (“forest fire” and “bushfire” are equivalent terms used in different parts of the world) smoke exposure is an emergency event which increases the challenge of timely, effective communications. Cowie et al. found that health communications during wildfire events are a major challenge; such communications lacked reference to health risk changes based on exposure level and ages, protective actions to limit exposure over periods of time, and effective reporting and dissemination pathways [2]. Walsh et al. found that caregivers of children aged 5–12 perceived smoke as a signal of wildfire danger rather than as a health hazard itself, and none of these caregivers had access to information about wildfire smoke intended to guard children’s health [1]. An additional issue is the general lack of alignment of wildfire smoke risk communication materials with accepted health literacy principles. Health literacy principles advocate for clear communication for the public; easy-to-understand materials to help people understand health information, make informed health decisions, and take health-promoting actions [39]. However, studies show that most health communications do not adhere to health literacy principles related to readability, accessibility, and actionability—a major problem for vulnerable populations [1, 36, 40, 41]. Although the fields of risk communication and crisis communication provide a wealth of evidence-based guidance on communicating general and emergency health risks [42, 43], wildfire smoke risk communication research has emerged mostly in the past decade and best practice guidance is still limited [13, 44].

### Objective of this scoping literature review

In 2021, members of our research team conducted an environmental scan [45] of existing and widely distributed wildfire smoke materials and determined that very few met the standards for good health literacy and clear communication. Given the gaps we found in our environmental scan, and the critical need for effective communication about wildfire smoke dangers and ways to reduce exposure, the need for a literature review in this area was clear. The objective of this scoping literature review is to identify: 1) relevant peer-reviewed studies about wildfire smoke risk communications, including communication resources for vulnerable, at-risk populations; 2) characteristics of effective communications, dissemination strategies, and gaps in the peer-reviewed literature; and 3) recommendations to improve wildfire smoke research and communication practices.

## Methods

### Inclusion criteria

This scoping review [46] focused on wildfire smoke risk communications for the public. Journal articles included in this scoping literature review were peer-reviewed, indexed in PubMed or Web of Science databases, available online, and available in English or Spanish. The search was not limited by years of publication.

### Exclusion criteria

Journal articles excluded were those focused on wildfires rather than wildfire smoke risk communications, for example, protecting one’s property, preparing one’s household for a fire (e.g., “go bags”), or escaping from wildfires. Articles about effects of fires on health outcomes, mortality-related studies, chemical fires, post-fire interviews, and smoke plumes with satellite/remote sensing were also excluded. Gray literature, general Internet searches, and case studies were excluded.

We excluded articles if indicated articles were not within the scope of our topic, based on title review, then conducted abstract review, and finally full article review/reading/abstraction. We also scanned bibliographies of articles to identify any additional articles that should be included.

### Search criteria

Article searches were conducted between October and December 2021 using PubMed and Web of Science databases. No limit was applied to the earliest date of publication to ensure the largest sample possible was collected. Search terms included: communication, communication strategies, education, effectiveness, environmental exposure, fire, risk communication, smoke, smoke exposure, wildfire smoke, and bushfire; various combinations of these terms were used. Bibliographies of articles were also reviewed for article inclusion.

### Process of review

After each search, three members of the research team (MV, SI, HB) reviewed the titles and abstracts of the outputs from the searches. After the initial title and abstract review, twenty-four articles were distributed to one of four team members (the three original team members and an additional member (LN)) for review. Throughout the period of review and abstraction, the team members met biweekly to address questions, discuss the articles, and talk through disagreements.

### Abstraction and analysis plan

**Fig. 1:**
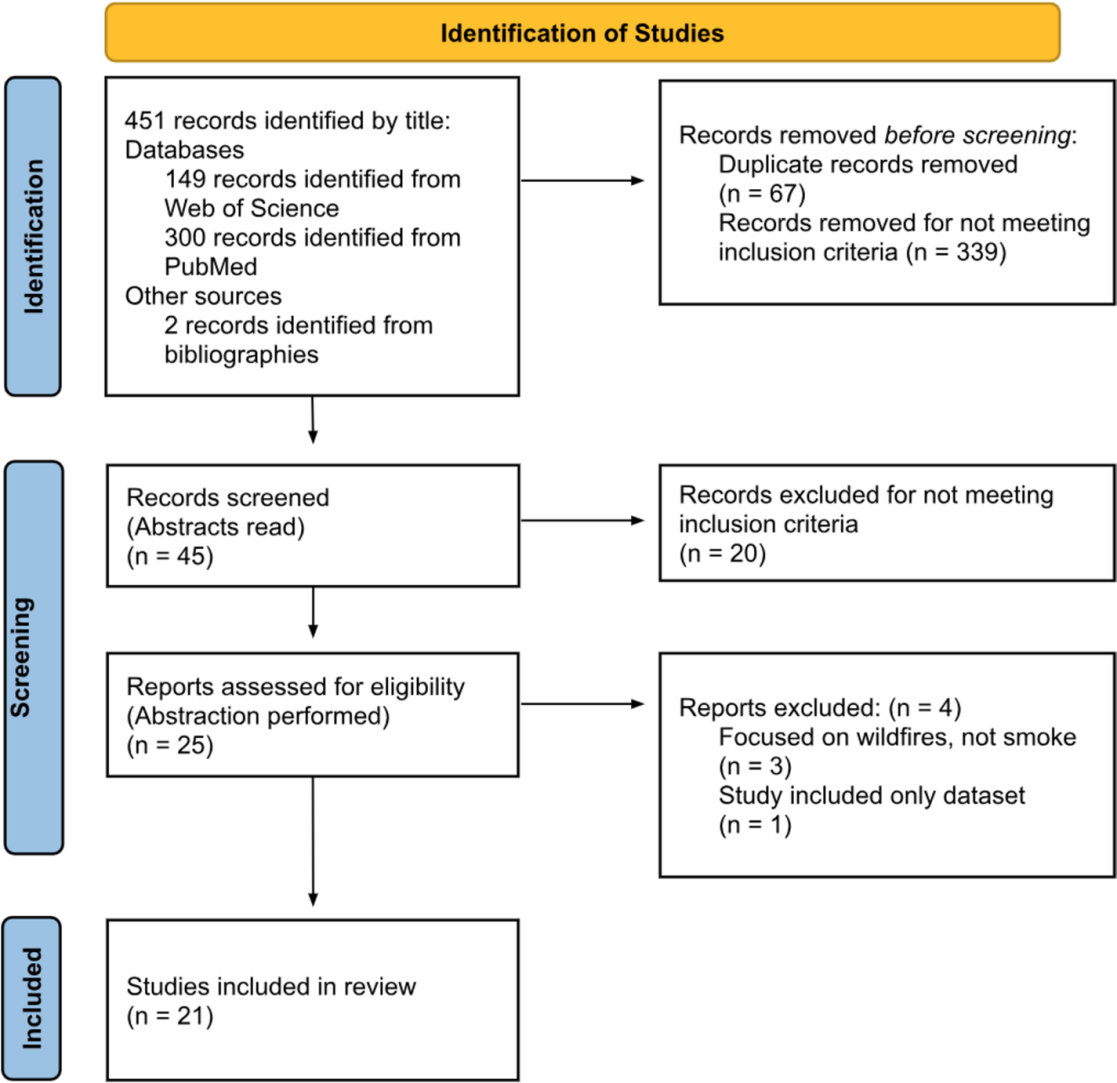
PRISMA diagram

The abstraction process included four categories for review: risk communication approaches, research design and methods, population in the study, and results and recommendations. These four categories were selected to ensure that data collected from articles were comprehensive, concise, and consistent. The category, “risk communication approaches”, organized information about risk communications programs, materials, and strategies. The category, “research design and methods”, was included to examine the quality and robustness of the articles. The category, “population in study”, provided context around population size, location, and sampling frame. The category, “results and recommendations”, contained key article findings and takeaways. After abstraction, the team determined that twenty-one articles would be included in the scoping review. A PRISMA Diagram depicts the flow of information (articles identified, included, excluded, and reasons) through the different phases of a review (see Fig. 1) [47].

Four members of the research team (MV, SI, LN, HB) conducted the qualitative thematic analysis. Data collected from articles during the abstraction process were coded and organized into categories. If category consistency was not reached, the research team discussed the issues and reached consensus. Analysis was an iterative process with additions or rearrangement of codes and categories. Themes were communication materials and messages, communication delivery strategies, behavior change, and communications for vulnerable populations. Categories were incorporated as subsections of the four themes. Google Workspace software [48] was used for this qualitative analysis.

### Quality of articles

Articles were assessed for quality based on study design and methods (see Table 1). Within the final sample, study designs, in order of rigor were randomized controlled trial, quantitative study, mixed methods study, literature review, and qualitative study. Authors reviewed papers for qualitative indicators, such as robustness of study design, recruitment, randomization, sampling, and methods (e.g., intervention, survey, database searches, interviews) with the intent of ranking them by rigor within each category. However, due to the limited sample of available articles and methodological similarities among multiple articles in each study design category, authors made the decision to organize articles alphabetically within each of the study design categories. Table 1 describes study design and methods.

**Table 1:** Summary of articles on wildfire smoke exposure risk communication approaches

Article	Location	Sample Description	Study Design	Key Findings
Randomized Controlled Trial				
Postma JM, et al. Promoting risk reduction among young adults with asthma during wildfire smoke: A feasibility study. 2022 [49].	Western US	*N* = 60 young adults (18–26 years old) with asthma diagnosis	Three-arm, unblinded, randomized controlled trial. Recruiting via university student listservs	- Smoke Sense app with features integrated by Urbanova was usable and acceptable by participants. Increased use of certain features (e.g. peer message boards) would improve app engagement.
Quantitative Study				
Cao Y, et al. Is a picture worth a thousand words? Evaluating the effectiveness of maps for delivering wildfire warning information. 2016 [50].	Western Australia	*N* = 261 residents of three fire-prone areas	Quantitative study. Pre-post experimental design. Online convenience survey, sampling from invitation postcards.	- Hybrid approach, combining maps (improved risk perceptions and comprehension) and text messages (describe locations e.g. shelters) was effective in communicating wildfire information.
Hano MC, et al. Knowing Your Audience: A Typology of Smoke Sense Participants to Inform Wildfire Smoke Health Risk Communication. 2020 [51].	United States	*N* = 5018 responses	Quantitative study. Post-intervention design. Individual level data from the Smoke Sense app	- Targeted approaches to risk communication should be based on participants’ perspective of smoke as a health risk.
Mott JA, et al. Wildland forest fire smoke: health effects and intervention evaluation, Hoopa, California, 1999. 2002 [52].	Humboldt County, California	*N* = 289 residents of the Hoopa Valley National Indian Reservation	Quantitative study. Retrospective design. Community survey of individuals treated at the reservation medical center and from randomly sampled households	- PSAs via radio/telephone reduced respiratory symptoms to wildfire smoke, PSAs + behavioral interventions are more effective, prioritize persons with pre-existing cardiopulmonary conditions, distribution of HEPA filters is effective.
Spano G, et al. Is Experience the Best Teacher? Knowledge, Perceptions, and Awareness of Wildfire Risk. 2021 [53].	Italy	*N* = 775 participants; *n* = 260 with direct wildfire experience, and *n* = 515 without direct wildfire experience	Quantitative study. Cross-sectional design. Single survey questionnaire comparing 2 groups.	- Those with direct fire-related experience had more knowledge of both wildfires and smoke exposure than those without direct experience. - Those with direct fire-related experience asked for more information about the topic of wildfires and smoke exposure.
Mixed Methods Study				
Chapple DR, et al. Communicating bushfire risk in the Blue Mountains: a case study of the Fire Stories film. 2017 [54].	Australia	*N* = 104 online questionnaires (84 from the cinema audience and 20 from the YouTube/DVD audience)	Mixed methods, quantitative and qualitative case study. Online survey from film audience database and other recruitment marketing	- Watching a video with real-life wildfire footage increased protective behaviors and concerns for wildfire smoke.
Sugerman DE, et al. Emergency Health Risk Communication During the 2007 San Diego Wildfires: Comprehension, Compliance, and Recall. 2012 [36].	San Diego, California	*N* = 1,802, those who received mass media messages during October 2007 wildfire	Mixed methods, quantitative and qualitative study. Random digit dialing survey from a landline database and census data	- Messages should include technical (e.g., masks) and non-technical (e.g., close windows) information. - Dissemination suggestions: TV, radio, newspaper, internet.
Literature Reviews				
Fish JA, et al. Effectiveness of public health messaging and communication channels during smoke events: A rapid systematic review. 2017 [44].	Varies, multiple locations	*N* = 10 studies included from North Rockies, South-Central US, Victoria Australia, Tasmania, New South Wales Australia, California, Montana, Oregon, South Carolina, North Carolina	Rapid systematic review of evidence check, qualitative, quantitative, and editorial papers	- Messaging should be clear, current, and consistent. - Dissemination strategies: TV, radio, local papers, road signs, interpersonal communications, etc. - Build on trust among neighbors and local residents and in communication channels and sources.
Heaney E, et al. Efficacy of Communication Techniques and Health Outcomes of Bushfire Smoke Exposure: A Scoping Review. 2021 [35].	Varies, multiple locations	*N* = 67 studies included from 20 Australia, 32 US, 8 Canada, 2 global, 1 of each: Southeast Asia, United Kingdom, Portugal, Sri Lanka, Belgium, China	Scoping review, PubMed and ProQuest databases	- Use messaging that is clear, current, and consistent. - Include information on locations, hazards and consequences, public-facing health and safety actions. - Tailor messaging to local contexts. - Combine traditional sources (e.g., TV) with non-traditional (e.g., social media). - Messages for at-risk populations should include actions to reduce wildfire smoke exposure.
Keegan SA, et al. Health protection messaging for populations susceptible to air pollution during landscape fire smoke events: an integrative review. 2021 [55].	Australia	*N* = 26 articles	Integrative review of articles related to health protection message pathways, databases (Medline, Scopus, Embase, and CINAHL) and gray literature search	- Deliver messages as early as possible, on both traditional (radio and television) and non-traditional (digital) information sources. - Collaboration between agencies is critical for future success.
Stieb DM, et al. Using maps to communicate environmental exposures and health risks: Review and best-practice recommendations. 2019 [37].	Canada	*N* = 37, 32 primary studies, 5 reviews/commentaries, half are in the US	Systematic literature review of commentaries, reviews, and primary studies (experimental, qualitative or mixed methods, cross-sectional)	- Map developers should engage with map users to develop maps. - Maps for risk communications need visual cognitive science (e.g. color, patterns) and actionable information.
Qualitative Study				
Burns R, et al. From hypothetical scenario to tragic reality: A salutary lesson in risk communication and the Victorian 2009 bushfires. 2010 [33].	Australia	*N* = 4 focus groups, 28 participants: landcare members (including fire volunteers), community health workers, two ‘new parents’ groups	Qualitative study. Brief questionnaire followed by focus group discussion of communications from different sources	- Include fire location, safety of situation, and contact information for evacuation centers. - Deliver information in local places and trusted local organizations (police, Red Cross) for those over 40 years old. - Deliver information via TV, word of mouth for those under 40 years old.
Damon SA, et al. Public communication in unplanned biomass burning events. 2010 [56].	Montana, Georgia	Sample size not available, participants in a break-out session	Qualitative analysis of interview/discussion hand-recorded notes. Verbal discussion in 90-min break-out session at a conference	- Annually update risk communications to maintain the public’s perception of risk. - Use ‘message blanketing’ at all local media outlets to promptly disseminate information.
Dodd W, et al. Lived experience of a record wildfire season in the Northwest Territories, Canada. 2018 [57].	Northwest Territories, Canada	*N* = 30 participants from Tribal Nations with 10 in Yellowknife, 7 in N’Dilo, 6 in Detah, 7 in Kakisa	Qualitative study. Semi-structured interviews with purposive sampling	- Community-based initiatives, including clearing flammable materials, increasing social support, and improving food security, can increase knowledge and lead to behavior change and reduce smoke exposure.
Errett NA, et al. Building a Practice-Based Research Agenda for Wildfire Smoke and Health: A Report of the 2018 Washington Wildfire Smoke Risk Communication Stakeholder Synthesis Symposium. 2019 [58].	Washington State, US	*N* = 76 practitioners and academics	Stakeholder synthesis, “World Cafe” meeting	- Government agencies and academic organizations desire more research related to smoke exposure, health risk, risk communications, behavior change and interventions, and legal and policy issues to increase the public’s knowledge of wildfire smoke risk.
Hano MC, et al. Scaling Up: Citizen Science Engagement and Impacts Beyond the Individual. 2020 [34].	Western US	*N* = 8 individuals, 3 employees of public organizations at the local level, 4 at the state level, and 1 at the tribal level in the western United States	Qualitative study. Semi-structured interviews for 45–60 min, with purposive sampling	- The Smoke Sense app is a good tool for individuals to use to increase knowledge about smoke risk to protect themselves and their families. - The app can be used on individual, organizational, and community levels for smoke risk communication. - More can be done for specific populations based on their unique needs.
Marfori MT, et al. Public Health Messaging During Extreme Smoke Events: Are We Hitting the Mark? 2020 [38].	Tasmania, Australia	*N* = 24 households in the Huron Valley	Qualitative study. Semi-structured interviews with convenience sampling	- Short and simple communication messages resulted in better recall. - Digital communications from public health and emergency services resulted in good recall yet also concerns about trustworthiness. - Communication about smoke was more often shared on social media compared to wildfire communications shared on other platforms.
Olsen CS, et al. Communicating About Smoke from Wildland Fire: Challenges and Opportunities for Managers. 2014 [59].	California, Montana, Oregon, South Carolina	*N* = 60, individuals in four research locations varying in geography, ecology, and social conditions	Case-study. Semi-structured interviews for 45–90 min, (key informants and small group) with purposive sampling	- Prioritize staff training in fire and smoke communications and outreach. - Coordinate messaging across agencies for better consistency, audience reach, and public trust.
Riden HE, et al. Wildfire Smoke Exposure: Awareness and Safety Responses in the Agricultural Workplace. 2020 [60].	San Joaquin, Imperial, and Salinas Valleys in California	*N* = 16 employers of farmworker *N* = 9 focus groups with 7–10 farmworkers each	Qualitative study. Semi-structured telephone interviews for 30–90 min and focus groups for 45–68 min Formative study.	- Safety precautions are limited for farmworkers. - Employers are unaware of safety guidelines to protect farmworkers. - Better resources are needed to assist employers and supervisors with complying to wildfire smoke safety regulations.
Thomas M, et al. Unpacking the Realities and Complexities of Sensemaking: Government Practitioners’ Experiences of Emergency Risk Communication. 2021 [61].	Victoria, Australia	*N* = 15 Emergency Risk Communication professionals	Qualitative study. Semi-structured interviews for 60 min, with purposive and snowball sampling	- Past experiences influence professionals’ abilities to make informed decisions during emergencies. - There is pressure for professionals to provide accurate information and during quickly moving time frames. - Making informed decisions was easier when working with the same set of stakeholders.
Van Deventer D, et al. Wildfire Smoke Risk Communication Efficacy: A Content Analysis of Washington State’s 2018 Statewide Smoke Event Public Health Messaging. 2020 [62].	Washington State, US	*N* = 273, message examples of wildfire smoke risk information from local and state government organizations and mainstream media in 8 counties	Qualitative content analysis. Purposive sample of counties, messages sampled from websites, deductive and qualitative methods.	- Wide variation exists in wildfire message content. - Improve coordination of information about health risks, smoke exposure reduction, and feedback from vulnerable populations.

## Results

The final sample of this scoping review consisted of twenty-one articles. Table 1 is a summary of the articles (see Table 1). Ten were based in the United States, six in Australia, two in Canada, and one in Italy. Two more articles included multiple countries in the studies. The results below are grouped into four sections related to communications materials, dissemination strategies, behavior change, and communications for vulnerable audiences—with a focus on gaps and recommendations. Table 2 contains detailed information extracted from the articles (see Table 2).

**Table 2:** Abstraction table of articles on wildfire smoke exposure risk communication approaches

**Article citation**	**Risk communication approaches**	**Research Design & Methods**	**Population in study**	**Results & Recommendations (listed)**
**Randomized Controlled Trial**				
Postma JM, et al. Promoting risk reduction among young adults with asthma during wildfire smoke: A feasibility study. 2022 [49].	The EPA Smoke Sense mobile application was adapted by technology partner, Urbanova. *SSU:* Participants recorded wildfire smoke observations, health symptoms, exposure reduction behaviors, and viewed information (air quality, next day forecast). *SSU-Plus:* Same features as *SSU* but had additional social features from Urbanova to maximize risk reduction (use of spirometer, mapping, and message board).	Three-arm, unblinded, randomized controlled trial. Recruiting via university student listservs. One control arm and two intervention arms using Smoke Sense Urbanova (SSU) and Smoke Sense Urbanova-Plus (SSU-Plus). Clinical outcomes were measured via Asthma Control Test (ACT) and forced expiratory volume (FEV). ACT measures five items (shortness of breath frequency, asthma symptoms, use of rescue medication, daily functioning, and asthma control) during self report recall. Higher scores indicate better control, with a score of over 19 indicating well-controlled asthma. FEV1 is the maximum amount of air that is subject can forcibly expel during the first–second following maximal inhalation. Decreases indicate airway narrowing. Spirometry measures FEV.	*N* = 67 young adults (18–26 years old) in Western US with self-reported asthma diagnosis by healthcare provider, own a smart phone, understand English. Most participants identified as female (79%), non-Hispanic (88%), and white (78%). Most reported using an iPhone (77%) versus Android (23%). At baseline the average ACT score was 20.4 (2.5), and the mean percent predicted FEV1 was 93.1% (17.8). Over half (55%) of participants reported being prescribed an asthma maintenance medication yet half reported ‘less than prescribed’ usage.	Smoke Sense app was usable and acceptable by intervention participants. In both intervention arms, *Smoke Sense* was primarily used to view the AQI and explore the fire and smoke map. *Smoke Sense* was less frequently used to report health symptoms, smoke observations and exposure reduction activities. And the least engagement was in learning risk reduction strategies. SSU-Plus had additional features, in order of frequency of use: spirometry, “Plus” menu of features (e.g., maps), and message board. 90% of SSU-Plus participants agreed that the app helped their asthma management, particularly with these features: air quality advisories, daily spirometry readings, and mapping feature, compared to 50% of SSU participants. SSU-Plus arm had significant increase in ACT at week 8 (Mean [SD]: 21.5 [2.3]) compared to baseline (20.0 [2.4]) (*p* = 0.0008), and significant decrease in percent predicted FEV1 at week 8 (88.6% [17.2]) compared to at baseline (94.9% [16.2]) (*p* = 0.0172). SSU arm had no difference in ACT at week 8 (21.0 [4.0]) from baseline (21.3 [2.1]) or in FEV1 at week 8 (95.6% [17.2]) from baseline (97.6% [14.6]) (all *p* > 0.05). Control arm had significant increase in ACT at week 8 (22.4 [1.9]) from week 0 (20.2 [3.7]) (*p* = 0.0320), but no change in percent predicted FEV1 at week 8 (92.9% [16.0]) compared to week 0 (88.4% [20.2]) (*p* > .05). - increased use of certain features (e.g. peer message boards) - weekly reminders to post messages - graph spirometry readings over time - more information about what spirometry readings meant would improve app engagement.
**Quantitative Study**				
Cao Y, et al. Is a picture worth a thousand words? Evaluating the effectiveness of maps for delivering wildfire warning information. 2016 [50].	Examination of the effectiveness of maps versus conventional text-based approaches for communicating spatial-related wildfire warning information in 3 fire-prone areas (urban, suburban and rural) of Western Australia related to residents’ abilities to use that information to predict fire spread and need for taking precautions. Experimental study based on understanding warning ‘effectiveness’ (warnings should appeal to general users, facilitate understanding and risk perception, assure information processing, and trigger appropriate responses) and effectiveness of maps for risk communication.	Online convenience survey recruiting via invitation postcards. Survey was conducted from Feb 2014 to Apr 2014 to quantitatively compare the communication outcomes of text-based messages versus various map designs and information elements (e.g., fire location, evacuation centers) within an experimental setting. Participants were tested on their abilities to extract key information about a simulated scenario of wildfire spread warnings from several different kinds of maps, and from text messages. They were also asked about their preference for maps versus text message information: i) accuracy of understanding, ii) risk perception, iii) response time, and iv) preference and ease of understanding.	*N* = 261 residents of 3 fire-prone areas (Kelmscott, Roleystone and Mundaring) in Western Australia. Sample was skewed towards residents who were middle aged (55% participants were 50–69 years old) and had higher education than general population of residents. 64% resided in place of residence for 10 or more years. 94% self-reported daily use of computers and 63% daily use of maps.	Accuracy of understanding, risk perception, response time, preference and perceived ease of understanding, and warning communications (map symbols, legends, text elements) were all measured by the survey. Significant differences (among different map designs: *p* < 0.05, *p* < 0.01, *p* < 0.005) were found between maps and text-based messages for understanding the wildfire risk situations. Map versions resulted in more accurate assessment of location, direction, and distance, and improved understanding of information than the text-based messages. Generally, when compared to text-based messages, maps stimulated better risk perception, but this varied between different communications. Response time also varied between maps and text-based messages, with some complex map designs resulting in a longer response time. Although maps did not always have better accuracy, risk perception, and response time, there was a preference for maps over text messages. Among 7 information elements, preference for text ranged from 1.2% to 37.1% of respondents, with majority of the respondents preferring maps or a combination of maps and text. Respondents reported better ease of understanding for all map designs except one (*p* < 0.005), compared to text. Warning communications included easily understood map symbols and colors, legends to explain color and size categories, and combining text elements with maps to improve descriptions. - utilizing specific map designs depending on the information to be delivered - incorporating visual maps and textual descriptions for enhanced understanding and interpretation
Hano MC, et al. Knowing Your Audience: A Typology of Smoke Sense Participants to Inform Wildfire Smoke Health Risk Communication. 2020 [51].	Assessment of user responses to the EPA Smoke Sense app, which is an educational mobile app about wildfire smoke exposure risks (including in specific geographic areas) and protective measures. The study objectives were to assess how user demographics, health status, information needs and risk perceptions were associated with preparedness to adopt health behavioral recommendations. Study aimed to identify specific clusters of traits related to intention to act (or not) and provide recommendations for risk communication for specific clusters of traits. Study further assessed how such traits map to several common health behavioral models, especially the Precaution Adoption Process Model.	Investigators used multiple cluster analysis to identify perspective 5 trait clusters based on users’ responses from Sept 2018 to May 2019 about their health status, experiences with wildfire smoke, risk perception, self-efficacy, access to exposure-reducing resources, health information needs, and openness to health risk messages. Differences in these traits were examined based on user demographics, health status, activity level and engagement level (from least to most engagement: Users, Explorers, Observers and Learners) with the app. Traits were mapped onto the Precaution Adoption Process Model relative to adopting recommended behaviors. Cluster findings were used to recommend health risk messaging users in each of the 5 clusters.	*N* = 5,018 participants in the US who provided information on the “citizen science” section of the Smoke Sense app. Respondents were mostly white (76%), college-educated (69%). 52% were female and 72% were between 30 and 65 years old. 92% reported “good” to “excellent” health. 72% reported wildfire smoke issues where they lived and most agreed such smoke was a health problem and risk could be modified by behaviors to reduce exposure. Respondents varied in their perceptions of information needs about wildfire smoke risk information.	Study findings identified 5 major traits based on respondents’ attributes and perceptions. Traits were not strongly identified by demographics, individual engagement with the health risk tool varied across traits. Traits were mapped onto the Precaution Adoption Process Model which indicated where the 5 trait clusters fell relevant to people’s intention to adopt recommended behaviors: “Unengaged”, “Susceptible”, “Proactive”, “Cautious”, “Protectors”. Health conditions, risk perception, resources, information needs, self-efficacy, and receptiveness to health risk communication all played a role in differentiating five traits. There were significant differences in how different traits engaged with the app at deeper Observer (ANOVA F-ratio: F (4, 4,777) = 8.242, (*p* < 0.005)) and Learner (F (4, 4,777) = 9.587, (*p* < 0.005)) engagement levels. Notably, the Susceptible trait, followed by Cautious trait, had the highest engagement as Observers and Learners. - tailoring risk messaging approaches for the 5 trait groups, including messaging for trait groups that may want more information, information about their smoke-relevant disease symptoms, information provided through health providers, etc. - further study of how the traits that emerged in the study can be used to guide more targeted risk messaging strategies.
Mott JA, et al. Wildland forest fire smoke: health effects and intervention evaluation, Hoopa, California, 1999. 2002 [52].	Interventions implemented by staff of the local medical center and other tribal organizations included distributing filtered and nonfiltered masks free of charge, vouchers for free hotel services in nearby towns to facilitate evacuation, portable high-efficiency particulate air (HEPA) cleaners, and several public service announcements (PSAs) through local media outlets. Due to limited resources, individuals who had adverse health effects and had preexisting conditions were prioritized for hotel vouchers and HEPA cleaners.	Retrospective quantitative study. A community survey of individuals treated at the reservation medical center and from randomly sampled households was completed. Individuals with preexisting cardiopulmonary conditions (coronary artery disease, asthma, chronic obstructive pulmonary disease, and other lung disease) were oversampled from the reservation medical center. And one randomly sampled person per household without any preexisting conditions was selected. The survey questions were about family demographics, intervention participation, and lower respiratory tract symptoms linked with forest fire smoke exposures before/during/after the large wildfire of 1999.	*N* = 289 residents of the Hoopa Valley National Indian Reservation, Humboldt County, California. Among subjects with preexisting conditions, 42.7% were male, 47.8% had household income at or below poverty level, 39.1% were less than 23 years old, 29.3% were 24–54 years old, and 31.5% were over 55 years old. Among subjects with no preexisting conditions, 49.2% were male, 55.3% had household income at or below poverty level, 35.4% were less than 23 years old, 52.8% were 24–54 years old, and 11.8% were over 55 years old.	Among evacuees of a wildfire smoke event, more than 80% of respondents correctly recalled a PSA without being shown a list of known PSAs, of these 66% took action to reduce smoke exposure as a result of hearing the PSA. Among those who could recall a PSA (*n* = 238), remaining indoors was the most common (78.6%, 95% CI: 73.4–83.8). Among those who took action because of a PSA (*n* = 157), “staying inside more often” was the most common action (83.4%, 95% CI: 77.6–89.2) taken. Respondents who recalled a PSA were less likely than those who could not recall a PSA to report worsening respiratory symptoms (OR = 0.25). - PSAs via radio/telephone reduced respiratory symptoms to wildfire smoke - PSAs combined with behavioral interventions are more effective - prioritize persons with pre-existing cardiopulmonary conditions.
Spano G, et al. Is Experience the Best Teacher? Knowledge, Perceptions, and Awareness of Wildfire Risk. 2021 [53].	Examine the impact of direct experience with wildfires on preparedness on the topic of wildfires among individuals. Assessing if there are group differences between fire-related employment or wildlife-urban interface proximity.	Cross-sectional design, single survey questionnaire administered throughout Italy. Questionnaire was disseminated through various channels, including social networks and personal and professional contacts snowball sampling. The general survey sample was grouped into two subsamples: (a) participants with direct wildfire experience and (b) participants without direct wildfire experience. Subsequently, the subsample of participants with direct experience was further classified as follows: (c) fire-related workers vs. (d) non-fire-related workers; and (e) WUI residents vs. (f) non-WUI residents.	*N* = 775 participants over the age of 18 and currently residents in Italy. Most respondents were from Apulia, (southern) Sardinia, Tuscany, (central) Lazio, and Lombardy (Northern Italy). Mean age was 37.4, and 50.7% female.	Individuals directly exposed to a wildfire, compared to those not exposed to a wildfire, have a significantly higher level of subjective knowledge of topic themes including wildfire definition (× 2 = 152.7, *p* < 0.001), self-perceived knowledge (× 2 = 17.2, *p* < 0.001), WUI definition (× 2 = 189.2, *p* < 0.001), and wildfire occurrence (× 2 = 23.4, *p* < 0.001), and significantly higher self-learning (× 2 = 0.3, *p* < 0.001) of the topic. There was no difference between the two groups on advanced knowledge, risk, and drivers/causes of wildfires. Participants with a direct experience of wildfires requested more information on the topic than those who had never been exposed to an event, which confirmed that knowledge and experience influence the number of sources from which individuals usually seek information. - need for developing effective communication for high-risk groups, such as homeowners and fire-related workers, in order to effectively prepare them for threats and potential impacts of wildfires, and avoid the adverse health impacts of the exposure.
**Mixed Methods Study**				
Chapple DR, et al. Communicating bushfire risk in the Blue Mountains: a case study of the Fire Stories film. 2017 [54].	Risk communication case study of a documentary film that was made as a tool for learning about natural disasters for many residents in the Blue Mountains who have little or no experience with bushfire due to the transient nature of these residents. The film was locally produced and used an event-based approach, using a past bushfire event to provide a vicarious experience, to demonstrate fire risk to motivate residents to be prepared for bushfires and to increase risk awareness.	Participants watched the film in 2013 (either at the cinema screenings or over YouTube/DVD) and were recruited from film audience database and promotions in newspaper and on social media, participated in a survey which was conducted over 4 weeks in April–May 2015. The survey sought to understand 1) what did they do to increase safety after viewing the film until the end of 2015, 2) how did they respond to the 2013 bushfire season in terms of safety-enhancing activity, 3) whether and to what extent did the film and other factors contribute to safety-enhancing activity, and 4) what aspects of the film contributed most to the safety-enhancing activity.	*N* = 104 online completed questionnaires: 84 from the cinema screening and 20 from YouTube/DVD audience. Participants lived in Australia. 66% lived in the upper Blue Mountains, 87% were homeowners, 57% female, 52% were over 60 years old.	Between watching the film and the 2013 fires, the cinema audience respondents reported 257 activities with 153 of them being “new” activities for bushfire preparedness activities (e.g., seek more information about risks, speak with household and community about risk information). 85% of respondents rated the film elther very effective or effective in promoting community preparedness and resilience. 71% rated the element of the graphic map of the movement of the fire as the most frequent “high impact”. 60% of respondents reported thinking about the film “for a long time afterward”. 72% of additional bushfire safety activity between viewing the film and the 2013 fire event were related to the film, newspaper articles, and the information exposition. There was a broadening of spheres of concerns after the film and before the fire with respondents indicating new concerns for their street, neighborhood, and someone nearby who needs help with respondents commonly crediting viewing the documentary for changes. The use of the film helped respondents understand the effects of the devastating 1957 fires, the implications of living in fire-prone areas, and the new actions to prepare for bushfire season. - use event-based approach for risk education to communicate, to enhance community bushfire safety efforts, to encourage social learning through storytelling,
Sugerman DE, et al. Emergency Health Risk Communication During the 2007 San Diego Wildfires: Comprehension, Compliance, and Recall. 2012 [36].	Evaluation of a study of wildfire risk communication mass media messages in San Diego County disseminated during the Oct 2007 wildfire. Messages were disseminated by the San Diego County Health and Human Services Agency and by the American Heart and Lung Association about ways to reduce exposure to wildfire smoke and ash. Messages in English and Spanish contained both “non-technical” (simpler actions: stay inside, close window, etc.) and “technical” (required access to products (home filtration, N85 masks, etc.) and/or performing more complex actions) recommendations. Messages were disseminated via TV, radio, newspapers and the internet over a 3 week period.	Random digit dialing survey from a landline database (no cell). English and Spanish respondents only above 18 years old. Questions adapted by a prior survey and pretested. Variables included: socio-demographic, exposure to wildfire smoke/ash, unprompted recall of messages, prompted recall of messages, communication source of messages, reported understanding of messages, reported actions taken, and whether respondent used inhaler/oxygen or sought medical care.	*N* = 1,802 survey respondents (48% response rate; 93% cooperation rate (completion/eligible) in San Diego, CA. 50% were male, and most were middle aged (35–64 years), non-Hispanic White, educated past high school, employed full time, and spoke English as their primary language.	Most persons surveyed reported hearing fire-related health messages (87.9%) and nearly all (97.9%) understood the messages they heard. Univariate analysis showed that compliance with most to all messages was higher among persons who were female, spoke English as their primary language, were educated beyond high school, and earned incomes of $50,000 or more (*p* < .05). Respondents complied with most to all of the nontechnical health messages, including staying inside the home (58.7%), avoiding outdoor exercise (88.4%), keeping windows and doors closed (75.8%), and wetting ash before cleanup (75.6%). In contrast, few (< 5%) recalled hearing technical messages to place air conditioners on recirculate, use High-Efficiency Particulate Air filters, or use N-95 respirators during ash cleanup, and less than 10% of all respondents followed these specific recommendations. The authors found that nontechnical message recall, understanding, and compliance were high during the wildfires, and reported recall and compliance with technical messages that required buying supplies (N85 masks, HEPA filters, or carrying out more complicated actions) were much lower. - limit the number of messages, explaining “technical” messages better - making messages more accessible to vulnerable populations - future disaster health communication should further explore barriers to recall and compliance with technical recommendations
**Literature Reviews**				
Fish JA, et al. Effectiveness of public health messaging and communication channels during smoke events: A rapid systematic review. 2017 [44].	Reviewed for these qualities Communication channels: mass media, interpersonal, road signs, presentations at public events. Effectiveness: awareness of public health messages, communication channel preferences, communication channel use, trust in communication sources, and compliance with advisories.	Rapid systematic review, search of 12 databases: MEDLINE, CINAHL, Current Content Connect, EMBASE, Science Direct, Scopus, PsycINFO, PsychARTICLES, JBI Databases of Systematic Reviews and Implementation Reports, Cochrane Database of Systematic Reviews, Evidence for Policy and Practice Information and Coordinating Centre/EPPI-Centre, the Campbell Library, and Database of Abstracts of Reviews of Effectiveness. Search terms included: “smoke event”, “bush fire”, “forest fire”, “wildfire”, “vegetation fire”, “wildland fire”, “prescribed fire”, “prescribed burn”; “public health messaging”, “risk communication”, “health warning”, “community warning”, “emergency warning”, “health information”. Study quality assessed based on effectiveness of communication channels for the general population, effectiveness of communication channels for at-risk populations, effectiveness of public health messages disseminated during smoke events. Articles from 2009 to 2016.	*N* = 10 articles from various geographic areas: North Rockies, South-Central US, Victoria Australia, Tasmania, NSW Australia, CA, Montana, Oregon, South Carolina, North Carolina. Review done by Australian research team.	Mass media, communication with healthcare workers, social networks from evidence are weak in terms of effectiveness of channels and sources of info, but indirect evidence shows these might be effective. Childcare, schools, retirement facilities are effective communication channels for specific age-defined groups. Neighbors and local residents were a trusted source of info. Preferred communication channels in US Australia: TV/radio/local paper, personal communication with authorities, social networks. Potential with texts, social media, mobile app, and twitter data, but insufficient evidence so far if communicating via internet is effective but may be useful depending on further research. - use multiple communication channels - leverage technologies such as social media and mobile apps - identify and use most effective communication challenges to reach at-risk and vulnerable communities - use simple language that is clear and specific about the situation with actions required - conduct evaluations and studies to strengthen evidence base of messaging interventions
Heaney E, et al. Efficacy of Communication Techniques and Health Outcomes of Bushfire Smoke Exposure: A Scoping Review. 2021 [35].	Papers covered media form and choice, smoke avoidance behaviors, advice on message style and content, communication limitations, bushfire smoke exposure, community medical/health seeking behaviors during smoke events, mortality and morbidity, psychological impacts, health consequences, health communication	Scoping review, search of PubMed database, ProQuest. Search terms included: “bushfire”, “prescribed burn”, “health messaging”, “wildfire”, “social media”. 20-year published time limit (Jan 1 2000 to Jun 1 2020).	*N* = 67 studies included: 20 Australia, 32 US, 8 Canada, 2 global, 1 of each: SE Asia, UK, Portugal, Sri Lanka, Belgium, China. Review done by Australian research team.	Communities used combo of traditional media sources (TV, radio, phone, newspaper) and non-traditional (Twitter and Facebook). Social media: real time dialogue between authorities and public, easy for messages to get amplified with sharing, builds trust when official accounts re-tweet or re-share posts. Still high utilization of traditional sources. Effective communication: guidance, timeframe, affected location, hazard/consequence, info source for those who want further info, guide public towards health promoting actions and safety during disaster, tailor info to local context to ensure info sources are credible/consistent, identify evacuation sites and any closed regions/roads. Messages: clear, specific, accurate, certain, and consistent, variety of languages and format, don’t inspire fear/panic in language, timely delivery, complete unbiased factual info, explain changes if any from prior messaging to keep consistent over time, encourage confidence, reduce confusion and distrust by ensuring consistent messaging. - Communication should use both traditional and non-traditional media sources. Also use different media at different stages of disaster to tailor info delivered to each individual. - Use effective communication and include pertinent info: actions/guidance, timeframes, affected location and evacuation areas, hazard, info source - Messages: clear, specific, accurate, consistent, objective, build public confidence and trust
Keegan SA, et al. Health protection messaging for populations susceptible to air pollution during landscape fire smoke events: an integrative review. 2021 [55].	Assess health protection messaging specific to smoke events, and risk communication contents surrounding bushfires and wildfires. Investigate which health protection message pathways and contents are appropriate for informing susceptible populations during smoke events.	Integrative review, searched Medline, Scopus, Embase, and CINAHL for peer-reviewed articles related to landscape fire air pollution and health risk communication. Search terms included: Bushfire, “bush fire”, wildfire, “wild fire”, “fire event”, “landscape fire”, “fire smoke”, “smoke pollution”, “air pollution”, “air quality”, “smoke event”; “Health protection message, “risk communication”, “public health message*”, “health warning”, “health alert”, “public health intervention”, “public health advi”, “health recommendation”. Also searched government websites, Google, Google Scholar, and websites for NGOs. Articles from 2010–2020, final search date was May 16, 2020. NOTE: articles aimed at assessment of interventions or strategies were excluded.	*N* = 26 articles included in final review. 15 US, 4 Canada, 1 Canada-US, 5 Australia, 1 multi-national. Review done by Australian research team.	Regarding content, messages were either 1) emergency health alerts or 2) information campaigns. Clear, short, and non-technical advice was more likely to be recalled and complied. Traditional and modern communication channels were being used effectively. There is some evidence that health alerts are less likely to be received by persons over 75. There was sparse information on susceptible populations, which stated the need for messaging to begin ahead of bushfire season. Regarding delivery, this was essential to build community trust. Inconsistent messaging was confusing and lacked referencing of trusted sources. Scant and somewhat conflicting evidence on susceptible populations’ use of information. Television is preferred source of information but increasing preference for online and smartphone-based communication methods. Improved interagency collaboration and communication could help with better delivery. - more detailed information on smoke and its health impacts for fire-prone communities, targeted messaging campaigns for communities who have high levels of exposure or fewer resources to take action - more consistent, timely messaging and greater dissemination of information on AQI and protection strategies - need for communications to start before bushfire seasons and advanced planning for messaging during smoke events, especially for susceptible populations
Stieb DM, et al. Using maps to communicate environmental exposures and health risks: Review and best-practice recommendations. 2019 [37].	Examine the effectiveness or utility of maps as environmental exposure including wildfire smoke and health risk communication tools, addressed the issue from the perspective of health literacy, environmental health literacy, or public health ethics.	Librarian searched Embase (1974 to 2018 February 26) and MEDLINE (1946 to February 21, 2018) for commentaries, reviews, and primary studies about the evaluation of maps or map features as exposure and health risk communication tools. Search terms included: map, GIS, spatial analysis, pollution, environmental health, air, health behavior, health education, literacy, community, population, communication, public health, risk.	*N* = 37 total: 5 reviews/commentaries, 32 primary studies (18 US, 2 UK, 4 Australia, 2 Belgium, 3 Canada, 1 Austria/France/Germany, 2 Germany). The most common design was experimental (*n* = 9), followed by qualitative or mixed methods (*n* = 8) and cross-sectional surveys (*n* = 6). Half of the studies (*n* = 16) were conducted in the US. Review done by Canadian research team.	Map developers and users engage to define purposes, planning, and goals of maps, iterative process. Consider visual cognitive science (how maps are processed cognitively, images and purposeful info, color, position, texture, motion, patterns, objects). Consider risk perception differences between experts/non. Risk communications: convey spatial dimension of exposure, inform personal, community, or political decision-making to mitigate risk, improve knowledge, persuade behavior change, increase confidence in authorities, build capacity for stakeholder engagement. Communications with general public: maps improve understanding of risks and information, better comfort and accuracy of understanding, better risk perception, more meaning, and maps often more preferred - understand the map developer’s societal role and mental model underlying map design - define, understand and iteratively engage with map users - define developers’ and users’ purposes - inform map design using key theoretical constructs - account for factors affecting risk perception - adhere to risk communication principles and cartographic best practices - avoid intentional and unintentional misrepresentation - consider environmental justice and public health ethics implications
**Qualitative Study**				
Burns R, et al. From hypothetical scenario to tragic reality: A salutary lesson in risk communication and the Victorian 2009 bushfires. 2010 [33].	Four example messages were created for different time periods. First message for day of blaze has immediate danger and preventive action regarding the fire. Other three messages contain health warnings. Third and fourth message include a few sentences about smoke safety. Messages were disseminated via local papers, radio, TV, and internet.	Qualitative focus groups using a hypothetical bushfire scenario based on previous fires. This was a part of a larger study on additional disaster scenarios, participants recruited for this bushfire scenario were from larger study. Two messages for two time periods (day of blaze, two days later) were adapted from actual examples to test communications and timing. Researchers gave demographic and fire risk perception surveys then asked for comments on trustworthiness of message source.	*N* = 28 participants, Australia. Four focus groups: landcare members (including fire volunteers), community health workers, two ‘new parents’ groups. 4 male and 24 female, ages 20 through 60 and over, with and without young children, resident in town and semi-rural settings.	Those over 40 preferred radio and local papers, and emergency services. Those under 40 preferred TV and local papers, and state and council spokespersons. Greatest perceived source for all was family, friends, neighbors. For one message that mentions smoke was sent shortly after the fire - thoughts: alarming and anxiety-provoking, inadequate knowledge without clear action steps, seemed sensational and not reliable source, message was news not information. Other message that includes smoke and sent after the fire: more reassuring than concerning, threat to children was a “scare thing”, not enough information, no action steps. - communicate via local places (post office, school) - include information about location of fire, population message pertains to, phone number to evacuation center - include safety of situation, phone number for regular updates, primary school should be information source
Damon SA, et al. Public communication in unplanned biomass burning events. 2010 [56].	Verbal discussion of panel participants during conference: tools for communicating respiratory risk from biomass burning, information to know about a particular wildfire incident to communicate effectively with affected populations, disseminating information in timely and useful manner, information to know about the audiences at risk (e.g., health vulnerabilities, geographic location, demographics) to communicate effectively, what doesn’t work	90-min break-out session at a conference. Instruments/questions to discuss were about: basic tools for communicating respiratory risk, basic information to know about an incident and about at-risk populations to communicate effectively with affected populations, how to share information in timely manner, what doesn’t work. Used interview/discussion interview, hand-recorded notes, analyzed results afterwards	*N* = Number of participants not available. Montana, Georgia. Attendees of the 2007 International Biomass Smoke Health Effects conference cosponsored by University of Montana and CDC that attended this break-out session.	Through the lens of Health Belief Model and Stages of Change Model: communications must be “new” to prevent public from sliding back to earlier stage of change and from having tempered perception of risk, people need to believe they have sufficient self-efficacy and able to overcome barriers to taking action, must be notified appropriately when to take action and if any smoke conditions change. Have profiles on affected populations available to assess need for low-literacy, non-English, compliance with recommendations, status on Stages of Change continuum. Federal and state resources should be connected with local agencies, community-based organizations, emergency response, and media. Mobile air monitors should be given to public, “Stay inside” typical recommendation could use research on indoor air filters to be more scientifically grounded. - materials must be “new” so public risk perception does not decrease after weathering previous years’ events - convince public of personal relevance of threat and ability to take recommended protective actions - research-based public messaging must be protective and non-alarming and be able to convince people of threat existence-their susceptibility-their ability to act - channels: message blanketing (all local media outlets), local opinion leaders and organizations
Dodd W, et al. Lived experience of a record wildfire season in the Northwest Territories, Canada. 2018 [57].	Explore the lived experience of individuals and communities affected by the 2014 wildfire season in the Northwest Territories of Canada, and to examine the impact of the wildfires and smoke on mental and emotional well-being, physical health, and livelihoods	Qualitative interviews were conducted during the period of October–December 2015 with residents of the four communities. A semi-structured interview guide was collaboratively developed by the research team. The audio portion from each video recording was manually transcribed. An iterative reflexive process was used to identify themes that emerged from the interviews. Coding was conducted within the QSR NVivo 11 software.	*N* = 30 participants (10 in Yellowknife; 7 in N’Dilo; 6 in Detah; 7 in Kakisa). Community members from four Subarctic communities residing in the Northwest Territories in Canada. The communities were Yellowkinfe, N’Dilo, Detah, and Kakisa. N’Dilo, Detah, and Kakisa are majority Dene (First Nation) communities while Yellowknife’s population includes both Indigenous (First Nations, Metis, Inuit) and non-Indigenous residents.	This paper is a qualitative paper about tribal experiences after summers with wildfires and shows the impacts that fires have had on mental health (personal and community isolation), decrease in physical health and activity due to the smoke limitations, and limits in cultural and “land-based” activities along with the decreased availability of country foods. These adverse experiences encouraged opportunities for community members to support and care for each other, and for several community-based initiatives to begin. A community-based program (FireSmart), initiated by the Government of Northwest Territories in Yellowknife and Kakisa, provided a workshop to encourage residents to remove brush and other flammable materials from around their houses. - expand community-based initiatives to reach more people and ensure broad participation - need for increased dialogue and education within their communities concerning the current impacts of climate change and how adaptation could mitigate these impacts
Errett NA, et al. Building a Practice-Based Research Agenda for Wildfire Smoke and Health: A Report of the 2018 Washington Wildfire Smoke Risk Communication Stakeholder Synthesis Symposium. 2019 [58].	Stakeholder synthesis “World Café” meeting to define a practice-based agenda for wildfire smoke research, interventions and policies for Washington State.	Purposively-selected Washington state practitioners and academics with relevant professional responsibilities or expertise in wildfire smoke and health engaged in small group discussions using the “World Café Method” to identify practice-relevant research needs related to wildfire smoke and health. 8 Table (2 tables per population) were set up to discuss issues relevant to 4 affected populations: workers, at-risk, susceptible populations, rural, and urban/suburban populations. Notes from each discussion were coded and qualitatively analyzed using a content analysis approach.	*N* = 76 Washington State practitioners and academics from state and local public health, environmental, non-profit, policy and tribal groups and a few stakeholders from federal government.	Participants identified research needs that were grouped into the following research topics: exposure science, health risk research, risk communication research, behavior change and interventions research, and legal and policy research. Many needs were identified within those topical areas, including need for more precise data about wildfire exposure, short-term, long-term and cumulative effects of wildfire smoke exposure, assessment of risk perceptions and best communication messages/channels/messengers, prioritization of the most effective and feasible interventions for specific groups, research on effectiveness of interventions, and development of government policy regulations for worker protection, building codes, etc. - additional research is needed to support risk assessment, risk communication, and risk management to protect communities and workers across Washington state from the growing threat of wildfire smoke - encourage researchers, practitioners, and funders to use proposed research topics to inform future research-practice collaborations and policy- and practice-relevant research
Hano MC, et al. Scaling Up: Citizen Science Engagement and Impacts Beyond the Individual. 2020 [34].	Explore the value of the EPA’s “citizen science” Smoke Sense app. The app provides educational information, has a forecasting feature for wildfire smoke, and integrates user input about wildfire locations, health symptoms (including subclinical and psychological, etc. symptoms. The study explored the perceived value of the app for individual, organizational and community use. The investigators provided background on the emerging use of “citizen science” projects to address complex health/social problems like wildfire smoke.	Qualitative study with semi-structured key informant interviews to explore the perceived value of the Smoke Sense app at individual, organizational and community levels. Recruited via purposive sampling strategy, drawing on list of organizations and individuals who had interacted with the Smoke Sense team as part of the citizen science project; inclusion criteria: (1) members of organizations in the health and environmental fields and thus part of the system that responds to smoke events; (2) individuals who engaged with Smoke Sense during its pilot year; and (3) individuals who worked in areas affected by smoke during the 2017 wildfire season. Inductive approach to inquiry beginning with case of interest and describe it with the purpose of synthesizing meaningful insights, data analysis used within-case inductive phenomenological thematic analysis	*N* = 8 respondents. Participants included three employees of public organizations at the local level, four at the state level, and one at the tribal level in the western United States, all of whose work intersects wildland fire and associated smoke, air quality, and public health.	This research investigated the motivations and expectations of organizational leaders across public-serving organizations who engage in citizen science projects regarding wildfire smoke and health. Results showed that participants significantly valued the app and its “citizen science” approach to collecting data, including novel information about wildfire smoke impacts from the public. Participants identified 3 motivations for using the app: 1. Individual: getting information to protect personal/family health; 2. organizational: using the app to support the work their organization does to get out information about wildfire smoke exposures, and to build relationships between federal, state, local, and tribal partners; 3. Community: support community efforts to protect sensitive groups. Participants thought Smoke Sense was an important decision-making tool at all these levels. - further research needed about the app over time to gather more information about its use, value, and ways to improve it - increase the impact of our citizen science research by supporting partners and scaling up the opportunities for engagement within projects from individuals to organizations, to advance the collective goal of addressing overarching complex social problems
Marfori MT, et al. Public Health Messaging During Extreme Smoke Events: Are We Hitting the Mark? 2020 [38].	Examine the effectiveness of PH communications about smoke with participants wanting simple messages from official sites, details about short term and long term effects, and having concerns about the smoke on their health. Understand (1) the level of concern about the impacts of smoke on well-being, (2) how information about smoke and health was received and understood, (3) if public health information influenced individual actions and behavior, and (4) the acceptability of using portable HEPA cleaners for managing poor indoor air quality during the wildfires.	Qualitative research with semi-structured interviews with 24 households in Tasmania following a severe smoke episode in 2019). Recruited from news media, radio, newspaper, social media feeds of public health services and regional community groups, and direct invites to current users of smartphone app AirRater. Inductive analysis; interviews conducted 2–3 weeks after the fire	*N* = 24 residents from 24 different households in Huon Valley region of Tasmania. 18 of 24 were female, ages ranging from parents of young children to retirees, several with had a health condition affected by smoke such as asthma, and several were pregnant. At higher risk from exposure to air pollution from wildfire smoke and in the intended target group for public health messaging.	Participants almost universally commented about experiencing personal concerns, such as negative physical, social, and psychological impacts, from living in smoke-affected areas over a prolonged period but some were more concerned about the wildfires than about the smoke. Participants all recalled receiving different information for smoke and health from different sources: media releases, web-based information, social media posts from public health and emergency services, informal sources such as friends and colleagues, and information on social media reshared by individuals, community groups, and agencies. Participants had more distrust in messages the received from social media as compared to government-sponsored messages. Participants also stated that they searched more actively for information about the fires and smoke information was more incidental so some people might have missed the smoke information. Participants recalled simple and understandable main messages, and most would use portable HEPA filters again with one primary motive to avoid symptoms caused by smoke. -use simple messages that can be verified by the government and repeat these messages for smoke exposure as the fire continues - specify short-term and long-term health effects and more detailed information - advice should be more timely and practical - better information about AQI data to increase the public’s understanding - specify which messaging was more important, since participants found it hard to decide between smoke or wildfire hazards
Olsen CS, et al. Communicating About Smoke from Wildland Fire: Challenges and Opportunities for Managers. 2014 [59].	Communications recommendations focused on (1) importance of consistent and coordinated smoke and communication management, (2) prioritizing and strategies to reach current and extended audiences, and (3) develop personal relationships with members of the affected public.	Case-study with 45–90 min semi-structured interviews (mostly 1:1 key informant, some small group) among individuals in 4 research locations to provide diversity in geography, ecology, and social conditions, as well as communication strategies, partnerships in smoke management, and experiences and challenges	*N* = 60 participants. California, Montana, Oregon, South Carolina. Purposive sample of critical managers to make decisions on smoke management or as key stakeholder engaged in fire and smoke management discussion.	Most common strategies to communicate to public: radio announcements, websites, hotlines, public meetings. Challenges to communicating about smoke: uncertainty about effectiveness of communication strategies (how to effectively communicate, do messages reach as many people as intended), confusion from inconsistent messages from different agencies (unclear messaging sometimes contradictory, connection to regulations where agencies can prescribe burns but public cannot), internal priorities about importance of communicating with stakeholders (priorities between people in organizations, some push for public involvement, some want to do only what is minimally required), improve relationships with public - making public communication an institutional priority - coordinating messages across and within agencies - utilize social networks and optimize resources - piggybacking off of existing communication programs: media, signage like billboards
Riden HE, et al. Wildfire Smoke Exposure: Awareness and Safety Responses in the Agricultural Workplace. 2020 [60].	Examination of wildfire smoke events and the impacts on health and safety for the agricultural industry workforce, the existing safety practices related to poor air quality from wildfire smoke, and the workplace social dynamics that impact safety.	Semi-structured interviews were conducted with agricultural employers and focus group discussions were held with farmworkers in three regions of California. Telephone interviews were conducted with employers. Focus groups were conducted with farmworkers in the 3 California regions. Telephone interviews were in English, 30–90 min, not recorded, detailed notes taken with keywords noted verbatim. Focus groups convened in-person, in Spanish conducted by a bicultural native Spanish speaker with extensive experience conducting focus groups with the agricultural community, 45–68 min, recorded and transcribed. Use of Atlas Ti, primary and secondary codes, themes, systematic analysis.	*N* = 16 agricultural employers (English) and 9 focus groups (*N* = 7–10 participants each) of farmworkers (Spanish-speaking) in 3 areas of California. Employers: San Joaquin Valley (*n* = 6), Imperial Valley (*n* = 4), and Salinas Valley (*n* = 6). Focus groups: convened in the San Joaquin Valley (*n* = 4), Imperial Valley (*n* = 2), and Salinas Valley (*n* = 3).	Qualitative study findings included: Agricultural employers had varying knowledge about and experience responding to poor air quality due to wildfire smoke. Respirators or masks were not mentioned as a potential protective measure when describing their safety practices. Farmworkers reported experiencing poor air quality due to wildfire smoke, although knowledge of safety precautions varied. Farmworkers reported employer and supervisors’ attitudes toward safety as having the greatest impact on the implementation of workplace safety measures. Employers and workers had variable knowledge about smoke risk and mitigation strategies. In particular, masks were not always raised as a protection for workers. - assignment of responsibility for ensuring a safe work environment; knowledge of existing safety precautions and sources for learning about current air quality conditions - tailor safety messaging about wildfire smoke, health promotion, and workplace safety strategies for employers and employees, recognizing roles and power imbalances in the occupational context - need for wildfire smoke exposure resources (i.e., how to find your air quality index or protect your workers) to assist employers and supervisors in their compliance with new emergency safety regulation
Thomas M, et al. Unpacking the Realities and Complexities of Sensemaking: Government Practitioners’ Experiences of Emergency Risk Communication. 2021 [61].	Exploration of lived experiences of government practitioners’ experiences of emergency risk communication related sensemaking during public health emergencies. Sensemaking is the concept in the fields of organizational studies and emergency response that is defined as a cognitive process that primarily takes place in an individual’s head and as a social process that is carried out and constructed through interaction.	Semi structured interviews to explore the lived experiences of the ERC professionals, specifically with questions about their experiences for smoke events that occurred in the last decade in Victoria, Australia. Focused on experiences *during* smoke events to evaluate sensemaking to positive outcomes. Recruited via purposeful sampling from the Environment Protection Authority Victoria and subsequent snowball recruitment based on interviewees’ recommendations of individuals at the EPA Victoria, Department of Health and Human Services, Country Fire Authority, Department of Environment, Land, Water and Planning, and Emergency Management Victoria. Conducted between June 2019-March 2020 face to face at the office of each participant. Approximately 60 min. Recorded and transcribed. Analyzed using thematic analysis.	*N* = 15 interviewees working as ERC professionals (ERC = Emergency Risk Communication). Victoria, Australia. 10 female, 5 male, several to 25+ years of experience in 8 with science expertise (public health, environmental health, air quality), 5 in communication (emergency, community engagement), 2 in operations (emergency and firefighting). Environment Protection Authority Victoria (*n* = 8), Department of Health and Human Services (*n* = 4), Country Fire Authority (*n* = 1), Department of Environment, Land, Water and Planning (*n* = 1), Emergency Management Victoria (*n* = 1)	Balancing achievable plausibility with expected accuracy - it is hard for practitioners to send out messages when they are first presented with information and they don’t want to provide inaccurate information. The pressure of making sense - practitioners feel pressure due to the time-critical aspect of the job as well as pressure from organizational and practical settings and have competing demands to meet. Professional expert identities and roles delimiting sense making - many interviewees expressed that their job titles and roles were important delimit areas for sense making, but it was hard if there were new roles like in the case of the health risk officer. Past lived experiences facilitate sensemaking - those with more lived experiences were viewed as more able to understand/assess the current situation. Personal relationships aiding collective sensemaking - working with the same group of stakeholders and decision makers over time was helpful in creating trust within the group for sensemaking. - communication during the early stages of an emergency should outline what the government knows, what they do not know, and what they are doing to address uncertainty. Communication should explain that advice is based on the current information, and is subject to change as new information is available - operations-based preparedness activities (e.g. field exercises, drills) to increase the knowledge of individuals involved, develop risk communication material, and build relationships between inter- and intra-organizational stakeholders - preparedness activities should involve a variety of scenarios and simulate the pressure practitioners experience when sensemaking for real-world ERC, incorporate scientific information, and include risk communication language discussions to decide on consistent wording
Van Deventer D, et al. Wildfire Smoke Risk Communication Efficacy: A Content Analysis of Washington State’s 2018 Statewide Smoke Event Public Health Messaging. 2020 [62].	Assessment of the types and content of the messages communicated by local authority figures, local government agencies, and media channels. Messages were assessed for information on health risk, personal interventions, administrative interventions, vulnerable populations, and trusted sources of information.	Content analysis of messaging examples of wildfire smoke risk information from local and state government organizations and mainstream media in 8 counties in Washington State in 2018. This was a purposive sample. Deductive, qualitative content analysis methods were used to create a codebook and to classify messages. The Extended Parallel Process Model (EPPM) risk communication framework was used.	*N* = 273 messages sampled from websites (either subpages or articles). 8 counties in Washington State (represents 57% of the state’s population) chosen based on historic wildfire smoke impact and representation of urban, rural, and suburban jurisdictions in 8 counties.	A total of 85 government sources and 188 media postings were analyzed. Of the 273 total messages, summary statistics were calculated. 71% of government sources and 66% of media sources contained information about the health risks of wildfire smoke, respectively. Of the messages containing risk information, 53% and 48% of government and media sources, respectively, contained information about personal interventions, whereas 33% of government sources and 20% of media sources contained information about administrative interventions. For government and media sources containing risk information and personal intervention information, the most common personal interventions were reducing activity (84% and 65%, respectively) and staying inside (78% and 83%, respectively). No confidence intervals were given. Authors reported that HEPA filtration was not mentioned by any site and evacuation was not mentioned on government sites. Overall, the majority of wildfire smoke messages from government sources communicated health risks in the 2018 Washington State wildfire smoke event. About half of the messages communicated information about personal interventions to reduce smoke exposure, suggesting that they contain messaging elements theorized by the EPPM to promote behavior change. However, the types of interventions suggested, and vulnerable populations addressed, varied widely across messages, indicating that government and news media would benefit from increased coordination of information about the health risks of smoke exposure, including approaches to exposure reduction, and/or risk communication tools, templates, and resources. - need input from more diverse populations including unhoused populations (for instance finding a clean air space that is open for unhoused) - need for people to be referred to trusted websites for additional information - increase coordination among government and news media about health risks of smoke exposure

### Communications materials and messaging findings and recommendations

Fifteen articles examined communications materials and messaging, and they offered recommendations including discussion of messaging content and design (5 articles), approaches to communications (2), use of a mobile application (3), use of real-life video footage (1), use of mapping for information distribution (2), effectiveness of risk communications (6), and communication gaps (2).

#### General messaging content and design

Five articles recommended messaging that uses simple, direct, clear, current, accurate, and specific language [33, 35, 38, 44, 55]. Authors commented that it is important that the communications are well-recalled, understood, and followed. This messaging should include information about fire locations, timeframes, safety, where to get additional updates and actions that people can take [33, 35]. Messages should also be timely, practical, consistent, and provide details about short-term and long-term health effects [38, 55]. Marfori et al. also noted that participants remembered short messages and gained knowledge but expressed interest in having added messages that were about both the short-term and long-term health risks of wildfire smoke [38].

Two articles provided more guidance for risk communications [36, 56]. Damon et al. called for ‘new’ materials to maintain public risk awareness year after year [56]. Messaging must be non-alarming and scientifically grounded. The public must be convinced of their self-efficacy to take recommended protective actions and must be notified to take actions against wildfire smoke exposure in a timely manner. Sugerman et al. discussed messaging that contains both non-technical actions, such as staying inside and closing windows, as well as technical actions, including use of home filtration devices or N95 masks [36]. They found that people’s non-technical recall, understanding, and compliance is high, but that technical messaging about High Efficiency Particulate Air (HEPA) filters and N-95 respirators should be better explained to the public.

#### Use of various media for communications

Three articles addressed the benefits and barriers of using the Smoke Sense mobile application, and assessed the knowledge gains from using the app [34, 49, 51]. Smoke Sense is a smartphone application, originally developed by the US EPA, that provides wildfire-related health risk resources and engages affected participants (“citizen scientists”) on wildfire smoke issues. Smoke Sense also shares information about daily air quality, maps of fire locations, and satellite images of smoke plumes [51]. In two different papers, Hano and their EPA colleagues concluded that it was a valuable app to provide general information about smoke risk for individuals, organizations, and communities, but that it needed improvement to provide population-specific information [34, 51]. Authors found that the Smoke Sense app can support organizations that can disseminate information about wildfire smoke, as well as support community efforts to protect sensitive and vulnerable groups [34, 49, 51].

From these qualitative and quantitative studies, recommendations for messaging included increasing individuals’ knowledge about smoke risks to protect themselves or their families, building self-efficacy for reducing exposure and actions to take, linking exposures to symptoms and cost-benefit relationships as well as information related to individuals’ personal concerns, and emphasizing the impact that reducing exposure may have on what individuals care about, e.g., maintaining good health and fitness [34, 51]. Furthermore, related to behavioral outcomes, an unblinded randomized controlled trial found that Smoke Sense app use among a specific vulnerable group – young adult participants with provider-diagnosed asthma – resulted in better asthma management during poor air quality days [49]. Postma et al. discussed the use of different features (e.g., peer message boards, daily spirometry readings, and air quality updates), which were integrated by technology partner Urbanova, within Smoke Sense, to increase engagement from participants and knowledge about smoke exposure/its effects on those with asthma [49].

One qualitative study found that showing real-life, vivid video footage of past wildfires could be a useful communication approach because it led to an increase in some desired behaviors, such as seeking knowledge, reducing exposure, finding more information about risk to home/area, and speaking with household and community members—actions linked to reducing exposure to wildfires and smoke [54].

#### Use of mapping for information dissemination

Studies by Stieb et al. and Cao et al. found that maps were effective for sharing information as well as increasing people’s knowledge about wildfire smoke and other environmental emergencies [37, 50]. Steib et al. researched the use of mapping health risks from natural hazard exposures (floods, wildfires, and contamination of air and water) as a risk communication technique and found that maps were better understood and interpreted than text [37]. The authors concluded that app and map developers should engage in an iterative process to design maps. Inclusion of visual cognitive science features (e.g., color, position, patterns, motion) and actionable information was important to adapt maps for effective risk communications. Cao et al. recommended a hybrid approach that combines maps with selected text message information [50]. Appropriately designed maps also better communicated wildfire and wildfire smoke warning information, improved comprehension, elevated risk perceptions, and increased appeal to the public. These maps were best complemented by textual descriptions of safe shelters, such as landmarks, names, and addresses.

Effectiveness of risk communications: Overall, as described above, eight articles [34, 36–38, 49–51, 54] in this category included assessments of effectiveness of risk communications for improving knowledge, behavior change, and health outcomes. Two articles noted that people effectively understood and recalled information from general messaging content and design [36, 38]. Six articles found that the use of various media, such as Smoke Sense app, video with real-life footage, and maps (especially maps that include text linked to spatial features), helped to improve knowledge, behavior change, and health outcomes [34, 37, 49–51, 54].

#### Gaps in communications

Two papers examined gaps in communications [60, 62]. Van Deventer et al. found wide variation in message content [62]. The most common personal interventions included reducing activity and staying inside during wildfire smoke events. However, regarding information specific to vulnerable populations, less than half of 85 government sources (47 social media-based messages, 38 digital articles from website) and 188 media messages (all digital articles from news sources’ websites) examined contained information about wildfire smoke. Approximately half contained a reference to a trusted source of information, but high-efficiency particulate air (HEPA) filtration was not mentioned at all. They concluded that government and news media would benefit from improved coordination of information about health risks of smoke exposure, approaches to reduce exposure, input from vulnerable populations, and risk communication tools, templates, and resources. Riden et al. discussed limitations in safety precautions for farmworkers during wildfire smoke, including the need for protective masks or respirators, air quality monitors, and changes to work schedules during events, and suggested that better resources are needed to assist employers and supervisors in complying with wildfire smoke safety regulations [60]. Agricultural employers varied in their knowledge and experience relative to responding to poor air quality caused by wildfire smoke.

### Communication delivery strategies: dissemination methods, pathways, and recommendations

Eight articles discussed communication delivery strategies, including traditional and digital communication strategies (7 articles), and allocation of resources within agencies specifically designated for communication and outreach measures (1 article).

#### Traditional and digital communications

Eight articles outlined common communication channels [33, 35, 36, 38, 44, 52, 55, 56]; three of these articles were literature reviews [35, 44, 55].

Authors recommended communicating through a combination of channels: radio, television, internet, social media, social networks, hotlines, mass media, local papers, and phone to effectively deliver knowledge and encourage behavior change [33, 35, 36, 44, 55, 59]. Burns et al. found that respondents older than 40 years of age tended to receive risk communications through emergency services and city councils, radio and local papers, and from members of local organizations or government [33]. However, in 2009 and 2010, respondents under 40 years of age most commonly used television, local papers, friends, family, and neighbors. More recently in 2020, Keegan et al. stated that traditional sources like television have been the preferred method of communication delivery but that a preference for online and smartphone-based communication has emerged among younger, female, and urban populations [55]. Additionally, authors recommended locations for communications, such as post offices, road signs, schools, retirement facilities, childcare areas, presentations at public events, and billboards [33, 44, 59]. They commented that communication from healthcare workers, texts, and social networks may be effective, but require further research.

Authors provided recommendations on timing for communications delivery. The review by Keegan et al. concluded that messages should be delivered as early as possible to give people time to plan and act in the case of a smoke event [55]. Damon et al. discussed disseminating information in a timely and effective manner and using “message blanketing” at all local media outlets, in addition to statements from local opinion leaders and organizations [56].

Articles included findings regarding the knowledge gain and recall of communications about wildfire smoke. Mott et al. discussed people’s recall of several interventions including public service announcements (PSAs) after a wildfire event and commented that participants more frequently recalled PSAs distributed via radio and by physician/clinical personnel [52]. In a qualitative study done by Marfori et al., participants recalled delivery strategies including media releases, digital information and social media posts from public health and emergency services, and word of mouth [38]. Participants reported that social media was a source that provided more information about *smoke* than the information on wildfires that dominated the other news platforms. Participants also shared that it was difficult to know which sources to rely on and that they trusted official communications from governments more than those shared on social media from non-government sources. Social media was found to allow real-time dialogue between authorities and the public, and amplified messages.

#### Resource allocation for wildfire smoke communications

Olsen et al. suggested prioritizing fire and smoke-related communications within agencies by allocating agency resources specifically for staff training in communication and outreach endeavors, and for coordinating messages across and within agencies [59]. Authors found that taking advantage of existing resources including informal social networks among the public, and building long-term relationships both between agencies as well as with the public were viewed as effective in distributing communications to audiences [44, 55, 59]. Olsen et al. warned against inconsistent messaging from different agencies and inadequate reach of messages [59]. Their recommendations included aligning internal priorities when communicating and building relationships with the public, as well as evaluating communications delivery to the intended number of people.

#### Effectiveness of risk communications

Overall, five articles in this category included assessments of effectiveness of risk communications for improving knowledge gains, behavior change, and health outcomes. These articles discussed traditional and digital communications that were effective for successful and timely dissemination of risk communications [33, 44, 52, 55, 59].

### Motivating behavior change: knowledge acquisition and trust building

Eleven articles discussed behavior change, including motivating public behavior change through community-level interventions (5 articles), increasing knowledge about wildfire smoke risk exposure among practitioners serving the public (3 articles), and examining public trust (3 articles). All eleven articles assessed behavior and behavior change (but not behavior intention) in the context of reducing wildfire smoke exposure.

#### Community-engaged interventions

Five articles described in-person intervention approaches associated with behavior change [34, 49, 52, 54, 57].

Authors of two articles discussed community-level interventions for Indigenous populations. In their qualitative study, Dodd et al. interviewed members of three Tribal Nations in Canada [57]. They found that community-based initiatives could reduce the impact of smoke exposure on physical, mental, and emotional well-being. These initiatives included removing flammable materials from around houses, joining community social support time at the community hall, and improving food security and connection to land-based activities (e.g., berry harvesting, fishing). Mott et al. conducted a retrospective study of Hoopa Tribe members affected by the 1999 wildfire in Humboldt County, California (USA) [52]. The study focused on recall of multiple community-focused interventions: effectiveness of free masks, free hotel services to shelter from the smoke area, high-efficiency particulate air (HEPA) air cleaners (distributed to individuals with pre-existing conditions), and PSAs [52]. PSAs were the most effective intervention. Mask use was associated with increased time outdoors and therefore increased exposure, but evacuation to hotels was not conducive to continuing employment as many residents worked in fire camps to fight forest fires. As a result of PSAs, the most common action taken was to stay indoors, rather than leave the area, use a mask, or use an air cleaner. Participants had highest recall of PSAs distributed via radio compared to all other sources, some of which included physicians, social networks, television, newspaper, and the Tribal Council. Additionally, those who recalled any of the PSAs were less likely to report worsening respiratory symptoms.

Hano et al., Hano et al., and Postma et al. indicated that the Smoke Sense application may support behavior change at multiple societal levels [34, 49, 51]. At the individual level, to protect health and increase awareness out of concern for health of self and family. At the organizational level, to advance organizational efforts in the area by using the app to distribute new tools and resources regarding smoke. At the community level, to increase awareness of connections between wildland fire, smoke, air quality, and health [34, 49].

In their case study, Chapple et al. found that study participants exhibited more protective behaviors and concerns for wildfire smoke after watching a video with real-life footage, compared to those who did not watch the video [54].

#### Knowledge among practitioners

Authors found that professionals with past experiences in wildfire-related work had significantly higher knowledge of wildfires/smoke exposure [53], had improved ability to make informed decisions in the face of an emergency [61], and better understood the current situation [61]. Spano et al. discussed that those with direct experience had, and also asked for, more information about these topics, indicating that practitioners/those experienced with wildfires can be an especially valuable source of knowledge for others [53]. Thomas et al. interviewed emergency risk communication professionals, who shared that working with the same group of people and stakeholders over long periods of time was helpful to the professionals to make informed, tailored decisions during smoke event emergencies [61]. Additionally, Errett et al. found that governmental agencies and academic organizations, participants at a state-wide wildfire smoke exposure symposium, proposed key research areas to increase the public’s knowledge of wildfire smoke risks [58]. They encouraged researchers and practitioners to study the following areas: smoke exposure, health risk, risk communications, behavior change and interventions, and legal and policy issues.

#### Public trust

Burns et al., Olsen et al., and Fish et al. all highlighted the necessity of trusted sources of information, the role of local agencies and governments, and the importance of communication sources [33, 44, 59]. Authors found that sources within the same social network influenced the value of fire and smoke messaging, and that neighbors and local residents were trusted sources of wildfire smoke communications [44, 59]. In three articles, authors discussed building public trust and improving message effectiveness through credible information sources [33], communication channels [44], and re-tweets or re-shares of social media posts between official accounts [35]. Examples of sources trusted by the public include authoritative local sources, the local police, as well as the Red Cross and State Departments of Health Services.

#### Effectiveness of risk communications

Overall, ten articles in this category included assessments of effectiveness of risk communications for improving knowledge, behavior change, and health outcomes. Five articles discussed community-engaged interventions that were associated with behavior change [34, 49, 52, 54, 57]. Two articles assessed ways to increase knowledge among practitioners and the public [53, 58]. Three articles suggested that effective communications included building public trust and using credible information sources [33, 44, 59].

### Communications for vulnerable populations

Literature regarding risk communications for vulnerable populations to reduce wildfire smoke exposure was limited – only six articles were found. In general, authors found that communication resources for vulnerable populations were limited. For example, in Keegan et al., of twenty-six health protection messaging communications included in their study, only nine were relevant to vulnerable populations [55]. Specific resources relevant to pediatric populations were also found to be lacking [35].

Culturally and linguistically diverse groups, those with hearing, vision, and mobility-related disabilities, those living in high smoke-risk geographic locations, those with pre-existing chronic illnesses, those who are children or older adults, and those who are pregnant may benefit from targeted health recommendations about wildfire smoke exposure and resources on prevention/mitigation strategies [35]. Authors recommended prioritizing communications for communities that have greater exposure to smoke events [55], including actions to reduce wildfire smoke exposure instead of only updates about wildfire smoke situations [35], and providing communications to at-risk groups before the general population [44]. Mott et al. and Dodd et al. emphasized the importance of community-level initiatives for Indigenous populations in northwest Canada and in Hoopa Valley, California, to increase awareness and behavioral change [52, 57].

Postma et al. found that among young adults ages 18–26 with asthma diagnosed by healthcare providers, the Smoke Sense mobile application use was effective in increasing air quality awareness knowledge and improving asthma management [49]. Specifically, young adult participants thought that the version of Smoke Sense with Urbanova’s integrated features for mapping smoke areas, air quality advisories, spirometry graphs, weekly reminders, and peer message boards, was easy to use.

Effectiveness of risk communications: Postma et al. noted that use of the Smoke Sense mobile application and its integrated features was effective to increase knowledge about air quality and wildfire smoke and positive behaviors to manage asthma [49]. Other articles in this category did not assess effectiveness of risk communications for vulnerable populations.

## Discussion

Wildfire smoke exposure is a serious public health challenge as wildfires are rapidly increasing in prevalence and intensity [3, 36, 51, 59, 62] and duration. Further, wildfire smoke exposure is an emergency event that adds challenges to providing timely, effective risk communications [52]. Wildfire smoke negatively impacts health in multiple ways, including over the long-term. This scoping review focused on peer-reviewed literature which evaluated wildfire smoke risk communications for public audiences, and identified gaps in the available literature, key communication issues, and recommendations for improvement. A total of 21 articles were included. We reviewed these studies in the following four thematic areas: 1) communications materials, messaging content and design, use of different media; 2) communication strategies, traditional and digital approaches, resource allocation for communications; 3) motivating behavior change, community-based interventions, knowledge acquisition among practitioners, public trust; and 4) communications for vulnerable populations. Although this review found that there are important assessments of the effectiveness of risk communications, most of those examined knowledge gains, rather than behavior intention, changes, and health outcomes.

### Gaps in the literature

We found that peer-reviewed literature about wildfire smoke risk communications is still very limited but has notably increased in the past decade as reducing exposure to wildfire smoke has become a priority public health issue. Because of the limited literature, there are gaps in all areas related to wildfire smoke communication. Encouragingly, available literature shows exploration of many important risk communication areas, including experimentation with messages, media, participatory design, community interventions, and an increasing focus on assessing effectiveness of materials and strategies in domains that include increasing knowledge, reinforcing positive behavior intention and changes, and ultimately leading to better health outcomes.

A major current gap in the literature relates to studies about communications created with and for vulnerable populations. This is an important finding of this review because at-risk, vulnerable populations are those most in need of effective communications. Some specific vulnerable populations include those who have low connectivity, are low-income, less educated, rural, and/or limited English proficient. Additional vulnerable populations include Black, Indigenous, and People of Color (BIPOC), outdoor workers, Deaf or Hard-of-Hearing people, pregnant women, children (especially those with asthma) and others with existing chronic respiratory, cardiovascular, or diabetes conditions [3, 7, 52, 62, 63]. The few articles related to vulnerable populations were helpful to point out the need to prioritize these populations, to provide more targeted health messaging and to increase community-based initiatives to develop more successful approaches to support vulnerable groups [3, 7, 28, 51]. However, specific messaging recommendations from these studies are still fairly generic. Another gap is that though some articles evaluated communications for accuracy, simplicity, and actionability, no articles explicitly examined health literacy—a central issue for high quality risk communications [33]. Finally, as in any relatively new research and practice area, there is a need for both more qualitative depth and context in the studies as well as quantitative rigor, including prospective research.

### Recommendations from this review

Despite the gaps in the literature, existing studies do provide rich information about known weaknesses of current wildfire smoke communications and note many recommendations to improve them. Many key issues with wildfire smoke risk communications have been identified in the existing literature. These include issues related to community-engaged design, timeliness, understandability, visual and textual elements (including mapping), selection of communication mediums, trusted messengers, actionability of recommendations, and point of contact dissemination strategies. Although many articles in this review included some assessment of effectiveness of risk communications, more work is needed in this area to examine and increase knowledge, encourage behavior intentions and change, and improve health outcomes.

Important recommendations were identified in each of the four thematic areas we focused on in this review as summarized here:

1. Communication materials and messages: Use evidence-based messages and multi-media (print, internet, mobile applications, maps, videos, PSAs, etc.); include clear, specific, actionable ways to reduce wildfire smoke exposure; improve explanations for technical messaging, improve mapping techniques, and provide details about short- and long-term health effects.
2. Delivery strategies: Simultaneously disseminate messaging through multiple local media outlets such as through television, radio, newspaper, the internet, and social media.
3. Behavior change: Provide more information through trusted intermediaries, such as health and public safety providers, to increase the public’s knowledge about wildfire smoke and motivate behavior change; maintain consistent messaging between agencies, and build on existing communications from public officials to increase public trust and timely action.
4. Communications for vulnerable populations: Prioritize targeted communications for specific at-risk groups; use community-engaged design and testing with these groups to ensure that messages are relevant to the group’s needs, and are clear, actionable, and use preferred communication mediums.

Guidance from prior studies of risk communications in other areas indicates that simple and clear communications adapted to specific cultures, languages, health literacy levels, and other factors are essential so that risk communication meets the needs of groups most at risk [64]. It is important that all wildfire smoke communications are created with attention to health literacy principles, which also would likely benefit vulnerable populations. Recommendations for future research on wildfire smoke communication include evaluating the differential effectiveness of various kinds of messages and media, studying communications tailored for the diverse US populations, and co-developing communications using health literacy principles with vulnerable populations.

### Strengths

This is a scoping review of the peer-reviewed literature about risk communications related to wildfire smoke exposure. Scoping reviews are helpful prior to planning a study, to identify gaps in research, to tailor a study or design interventions, and to include multiple types of studies, not limited to randomized trials or quantitative studies. This can be especially important when there are few articles for unique populations, as we found in this review. There was no publication date limit selected to maximize collection of relevant articles. This scoping review also included articles from authors located in various countries affected by wildfire smoke.

### Limitations

There was a limited number of articles from any one country, limiting the ability to make any country-specific conclusions. Available articles in languages other than English and Spanish were not included, given our a priori inclusion criteria. No studies were found in Spanish language which may indicate a critical gap in the evaluation of wildfire communications for Spanish-speaking populations. The biggest limitation was the limited literature in this area to date, which impacted interpreting the issues and recommendations in this review. However, we did note that there was often convergence among authors about important issues cited and recommendations for improved wildfire smoke risk communications.

## Conclusion

Wildfire smoke exposure, with its detrimental effects on health, is a significant public health concern [3]. Given the growing magnitude of wildfire smoke exposure, there is a pressing need for more intervention and evaluation research about effectiveness of risk communications in this topical area. This scoping review examined peer-reviewed literature on wildfire smoke risk communication and identified gaps for additional research. Our review found limited studies describing characteristics of effective communication materials and messaging, and communication delivery strategies, and approaches to increase positive behavior intention, behavior change to reduce wildfire smoke exposure and, ultimately, improve health outcomes. Literature on these topics for vulnerable populations was even more limited. Although studies in the topical area of wildfire smoke messaging are still nascent, they provide important guidance for researchers and practitioners in developing and disseminating risk communications for the general public and for vulnerable populations.

Priority recommendations are that risk communications should be easy-to-understand, provide simple and direct messages, highlight specific actions to undertake to avoid smoke exposure, use mapping when relevant, use hybrid mixtures of formats (such as video, textual information, maps, mobile apps, etc.), and be customized to the needs of at-risk, vulnerable populations. These recommendations are similar to those for improving the quality and effectiveness of health communications in general. However, wildfire smoke exposure is also an emergency event that presents the added critical challenge of providing timely, effective communications from government and local community organizations. There is a lack of rigorous evaluation studies to demonstrate which communication strategies are most effective. This review could be augmented with a future review of studies in languages other than English and Spanish. The recommendations also propose using a wide variety of dissemination strategies relevant to the focal populations.

## Data Availability

All data generated or analyzed during this study are included in this published article [and its tables and figure].

## Abbreviations

WUI: Wildland urban interface
BIPOC: Black, Indigenous, and People of Color
EPA: Environmental Protection Agency
HEPA: High-efficiency particulate air
PSAs: Public service announcements
